# Effects of publicly funded and quality of life on attendance rate among methadone maintenance treatment patients in Taiwan: an 18-month follow-up study

**DOI:** 10.1186/s12954-015-0076-8

**Published:** 2015-10-16

**Authors:** Kun-Chia Chang, Chung-Ying Lin

**Affiliations:** Department of General Psychiatry, Jianan Psychiatric Center, Ministry of Health and Welfare, Tainan, Taiwan; Department of Public Health, College of Medicine, National Cheng Kung University, 1 University Road, Tainan, 70101 Taiwan

**Keywords:** Methadone maintenance treatment, Publicly funded, Quality of life, Self-paid, Substance abuse

## Abstract

**Background:**

Methadone maintenance treatment programs (MMTPs) are important public health intervention to control the human immunodeficiency virus (HIV) and the drug use problems. For expanding treatment coverage, publicly funded programs may be necessary for heroin users with low socio-economic status. We evaluated the difference of demographics, clinical features, and quality of life (QoL) of heroin users enrolled in publicly funded and self-paid MMTP and explored determinants influencing their attendance rate, respectively, for these two groups.

**Methods:**

A total of 234 heroin users enrolled in MMTP (129 in publicly funded and 105 in self-paid) between 2006 and 2008 self-reported the Taiwan version of the World Health Organization Quality of Life Instrument, Brief Version (WHOQOL-BREF) at baseline. Data regarding demographic and clinical features were collected during baseline interview. Methadone per 3-month attendance rates up to 18 months were conducted for each participant beginning from the index date.

**Results:**

Self-paid group had a better QoL but lower treatment adherence than did the publicly funded group. Male and living alone were positive predictors on attendance rate for publicly funded group, and age of first heroin use and hepatitis C virus (HCV) seropositive were negative predictors. However, predictors on attendance rate for self-paid group were different from publicly funded group: HCV seropositive was a positive predictor and social QoL was a negative predictor.

**Conclusions:**

Findings of this study should be concerned with modifying original funding eligibility. Additional measures to explore what could impede treatment adherence are needed.

## Background

Taiwan implemented a National Health Insurance (NHI) program from March 1995, offering a comprehensive, unified, and universal health insurance program to all citizens [1]. The coverage includes outpatient service, inpatient care, Chinese medicine, dental care, childbirth, physical therapy, preventive health care, home care, and rehabilitation for chronic mental illness. In addition, the NHI program covers as high as 99 % of Taiwan population [2]. However, the cost on items directly related to substance use, such as the methadone maintenance treatment (MMT), is not covered by the NHI. MMT is a primary treatment for opioid-dependent people who have significantly elevated mortality than their general counterparts [3], and opioid dependence makes global disease a burden [4]. Although numerous studies [5–9] report that MMT has encouraging results of reducing drug addiction, decreasing illegal activities, and improving the overall well-being for drug users, the MMT program (MMTP) was not permitted until 2006: The Taiwan Center for Disease Control (CDC) permitted MMTP for patients with opioid dependence in response to human immunodeficiency virus (HIV) epidemic [10]. Several publicly funded MMTPs were held due to the urge for inhibition of the HIV infection spread. The criteria of participating in a publicly funded MMTP were that the heroin users were HIV seropositive, ex-prisoners, or applying for deferred prosecution. The publicly funded MMTP provided free treatment for heroin users with HIV seropositive and 1-year service without payment for those who applied for deferred prosecution or who were ex-prisoners. However, those heroin users who did not fulfill the publicly funded criteria were charged around 100 US$ per month for the treatment (self-paid MMTP). Previous American studies [11, 12] found that Medicaid eligibility was strongly associated with enrollment in MMTP, and Medicaid clients had far greater access to MMTP than non-Medicaid counterparts after controlling their characteristic differences. However, studies with regard to differences between publicly funded and self-paid MMTP have been minimally addressed, especially in the East.

As for comparing the publicly funded and self-paid MMTP, we recommend using an important outcome index, quality of life (QoL). QoL refers to a subjective evaluation which is embedded in a cultural, social, and environmental context [13]. There were increasing evidences that a good QoL is associated with a better treatment outcome in patients with opioid dependence, e.g., [14, 16]. In addition, research has found that co-morbid infectious disease such as HIV and hepatitis C virus (HCV) and drug-related crimes which are frequent in heroin users may impair their QoL [15–17]. Therefore, understanding the QoL and features of the two MMTP groups may provide clinicians useful information and help them make a critical clinical decision.

In addition to the QoL, adherence is another important issue for MMTP participants. Several studies from the West [18–21] found that high methadone adherence is necessary for successful therapeutic outcomes. Furthermore, these studies [18–21] used retention rate as an index to represent the adherence. However, if the policy provides no take-home dose for methadone maintenance patients (e.g., the MMTP in Taiwan), we suggested that treatment attendance, another adherence index, becomes essential for understanding treatment effectiveness. Successful MMT requires both long-term enrollment (retention duration) at an adequate dosage and on daily basis (regular attendance) [9]. In addition, to the best of our knowledge, studies reporting the factors influencing on methadone adherence in Asia are still scarce. Therefore, investigating the factors that influence on MMTP attendance rate in an East-Asian country (say, Taiwan) is necessary.

Using an 18-month follow-up data from one psychiatric center in south Taiwan, this observational study aimed to (1) examine the QoL difference between methadone maintenance patients presented for publicly funded and self-paid MMTP and (2) explore determinants influencing MMTP adherence using attendance rate, respectively, for these two groups.

## Methods

### Data sources

A retrospective cohort study was conducted, and all recruited participants between March 2006 and July 2008 were diagnosed as opioid dependence by a board-certified psychiatrist from the Jianan Psychiatric Center research team. The inclusion criteria were as follows: (1) being more than 20 years old; (2) meeting the DSM-IV (*Diagnostic and statistical manual of mental disorders, fourth edition*) criteria for opioid dependence; (3) having sufficient mental competence to understand and sign an informed consent; (4) residing near our treatment service site (i.e., Tainan City); and (5) no other MMTP contraindication, such as severe liver cirrhosis, severe cognitive impairment, or behavioral disturbances. None of the participants had participated in any MMTP before the intake interview. At the intake interview, the participants were identified as publicly funded MMTP (*n* = 129) or self-paid MMTP (*n* = 105) groups based on whether they met the publicly funded eligibility criteria: (a) as a function of HIV status; (b) applying for deferred prosecution; and (c) had been incarcerated due to the Drug Act before and discharged during the recruited period. Methadone attendance of 18 months was conducted for each participant from the index date, and we divided the study period into six phases of 3 months duration each. The Hospital Ethics Committee of Jianan Psychiatric Center approved this study (IRB number, JMH9601).

### Quality of life

We used the World Health Organization Quality of Life Assessment, Brief Version (WHOQOL-BREF), which contains 28 items with 26 standard items from the original WHOQOL-BREF, and two Taiwanese national items [22]. In addition, the WHOQOL-BREF Taiwan version includes four domains (physical, seven items; psychological, six items; social, four items, and environment, nine items) and two generic items which did not belong to any domain (“overall QoL” and “general health”). Domain scores are calculated into a 4-to-20 range, and a higher score represents a better QoL. Moreover, the psychometric properties of WHOQOL-BREF are satisfactory in Taiwan population [22], including those with a mental illness [23–25].

### Demographics and lab tests

In addition to the WHOQOL-BREF, each MMTP patient completed a background information sheet including his or her birth date, gender, living status (alone vs. with others), educational years, age of first heroin use, heroin using years, employment status (fixed or not), and family drug using. After completing the WHOQOL-BREF and the background information sheet, each patient underwent a series of laboratory tests, including HIV, HBV, and HCV tests.

### Data analysis

Baseline characteristics and QoL scores between publicly funded and self-paid MMTP groups were compared using *χ*^2^ tests (for frequency comparisons) and independent *t* tests (for mean comparisons). In addition, QoL scores of the two groups were separately compared with QoL scores of Taiwan population using one-sample *t* tests. The mean (±SD) QoL scores of Taiwan population are 14.06 ± 2.34 for the physical, 13.23 ± 2.15 for the psychological, 13.56 ± 2.29 for the social, and 12.72 ± 2.07 for the environment domains [26].

Generalized estimating equation (GEE) was used to examine the effects of several predictors on attendance rate. Because each participant had one to six attendance rates (i.e., the attendance rates for 3, 6, 9, 12, 15, and 18 months) from participating in the MMT program to 18 months later, using the GEE is adequate. All the analyses were done using SPSS 15.0 (SPSS Inc., Chicago, IL.).

## Results

No significant differences were found between the publicly funded and self-paid MMTP participants in their demographic data, except for heroin using years (*t* = 3.59, *P* < 0.001) and HIV carrier (*χ*^2^ = 45.35, *P* < 0.001). The publicly funded group had significantly more heroin using years (9.25 ± 6.77 vs. 6.48 ± 3.86) and HIV carriers (*n* = 45 vs. 0) than the self-paid group. In addition, the self-paid MMTP participants had significantly higher QoL scores than the publicly funded MMTP participants in all QoL domains (Table 1).

**Table 1:** The baseline characteristics and QoL scores comparisons using independent *t* tests (for continuous variables) or *χ* ^2^ tests (for categorical variables) between publicly funded and self-paid methadone maintenance treatment patients

	Publicly funded		Self-paid		*χ* ^2^ or *t*	*P*
	*n*	Mean (SD)	*n*	Mean (SD)		
Age (year)	129	38.29 (7.65)	105	37.97 (7.16)	0.32	0.747
Gender					0.93	0.334
Female	11		13			
Male	118		92			
Living alone^a^					1.99	0.158
No	118		98			
Yes	11		4			
Educational years	129	9.36 (2.55)	105	9.48 (2.09)	0.40	0.688
Age of first heroin use	129	25.59 (5.89)	105	26.92 (6.80)	1.65	0.101
Heroin using years^a^	112	9.25 (6.77)	82	6.48 (3.86)	3.59	<0.001
Fixed employment^a^					<0.01	0.975
No	60		49			
Yes	68		56			
Family drug using					0.47	0.495
No	109		92			
Yes	20		13			
HBV carrier					0.28	0.597
Seronegative	106		89			
Seropositive	23		16			
HCV carrier					3.30	0.069
Seronegative	4		9			
Seropositive	125		96			
HIV carrier					45.35	<0.001
Seronegative	84		105			
Seropositive	45		0			
Physical QoL^a^	128	11.95 (1.89)	103	12.48 (1.94)	2.10	0.036
Psychological QoL	129	11.94 (2.68)	105	12.83 (2.12)	2.83	0.005
Social QoL^a^	129	12.62 (3.12)	104	13.44 (2.80)	2.09	0.038
Environment QoL	129	12.47 (2.86)	105	13.43 (2.30)	2.77	0.006

*QoL* quality of life, *HBV* hepatitis B virus, *HCV* hepatitis C virus, *HIV* human immunodeficiency virus

^a^With missing values

As compared with Taiwan population, the publicly funded MMTP participants had all but the environment domain scores significantly lower than the scores of Taiwan population. In addition, the self-paid MMTP participants had significantly lower QoL score in the physical domain and higher score in the environment domain (Table 2).

**Table 2:** Comparisons using one-sample *t* tests between methadone maintenance treatment patients and Taiwan population

		Publicly funded	Self-paid
Physical QoL	*n*	128	103
	Mean (SD)	11.95 (1.89)	12.48 (1.94)
	*t* (*P*)	−12.66 (<0.001)^a^	−8.25 (<0.001)^a^
Psychological QoL	*n*	129	105
	Mean (SD)	11.94 (2.68)	12.83 (2.12)
	*t* (*P*)	−5.45 (<0.001)^b^	−1.93 (0.056)^b^
Social QoL	*n*	129	104
	Mean (SD)	12.62 (3.12)	13.44 (2.80)
	*t* (*P*)	−3.42 (0.001)^c^	−0.43 (0.669)^c^
Environment QoL	*n*	129	105
	Mean (SD)	12.47 (2.86)	13.43 (2.30)
	*t* (*P*)	−0.98 (0.327)^d^	3.15 (0.002)^d^

^a^Compare with Taiwan population (mean ± SD = 14.06 ± 2.34)

^b^Compare with Taiwan population (mean ± SD = 13.23 ± 2.15)

^c^Compare with Taiwan population (mean ± SD = 13.56 ± 2.29)

^d^Compare with Taiwan population (mean ± SD = 12.72 ± 2.07)

Different predictors of attendance rate were found between publicly funded and self-paid groups. Significant predictors for the publicly funded group were gender (reference = female; *β* = −0.115, *P* < 0.05), living alone (reference = no; *β* = 0.088, *P* < 0.01), age of first heroin use (*β* = −0.007, *P* < 0.05), and HCV carriers (reference = seronegative; *β* = −0.068, *P* < 0.05). As for the self-paid group, significant predictors were HCV carriers (reference = seronegative; *β* = 0.224, *P* < 0.01) and social QoL scores (*β* = −0.020, *P* < 0.05) (Table 3).

**Table 3:** Predictors of attendance rate on the heroin-dependent individuals receiving methadone maintenance treatment using generalized estimating equation models

	Attendance rate (3, 6, 9, 12, 15, and 18 months)			
	Publicly funded		Self-paid	
	*β*	(95 % CI)	*β*	(95 % CI)
Age (year)	0.005	(−0.001, 0.010)	0.001	(−0.004, 0.006)
Gender (ref: female)	−0.115*	(−0.216, −0.013)	−0.017	(−0.101, 0.066)
Living alone (ref: no)	0.088**	(0.024, 0.152)	0.023	(−0.111, 0.156)
Educational years	−0.005	(−0.001, 0.010)	−0.013	(−0.026, <0.001)
Age of first heroin use	−0.007*	(−0.012, −0.001)	<0.001	(−0.006, 0.005)
Fixed employment (ref: no)	−0.002	(−0.055, 0.051)	0.053	(−0.004, 0.111)
Family drug using (ref: no)	0.046	(−0.016, 0.107)	0.026	(−0.074, 0.125)
HBV (ref: seronegative)	−0.015	(−0.093, 0.063)	0.026	(−0.062, 0.113)
HCV (ref: seronegative)	−0.068*	(−0.120, −0.016)	0.224**	(0.085, 0.363)
HIV (ref: seronegative)	0.009	(−0.051, 0.069)	–^a^	–^a^
Physical QoL	−0.015	(−0.034, 0.004)	0.022	(−0.001, 0.045)
Psychological QoL	−0.010	(−0.025, 0.006)	−0.011	(−0.031, 0.009)
Social QoL	−0.002	(−0.017, 0.013)	−0.020*	(−0.034, −0.006)
Environment QoL	0.012	(−0.003, 0.028)	0.019	(−0.001, 0.039)

*QoL* quality of life, *HBV* hepatitis B virus, *HCV* hepatitis C virus, *HIV* human immunodeficiency virus

*P < 0.05, **P < 0.01, ***P < 0.001

^a^No values due to no HIV patients in this model

## Discussion

Our results are consistent with previous studies: heroin users had lower QoL scores as compared with the general population [27]. This also echoes the findings in other research that heroin users often find themselves in a crisis situation at the MMTP intake and attend treatment in poor condition, resulting in low QoL scores at admission [28, 29]. However, unlike the publicly funded group who had all domains of QoL but environment QoL significantly lower than the Taiwan general population, the self-paid group only had its physical QoL lower than the Taiwan general population. One possible explanation is that the eligibility of free treatment covered heroin users who contracted HIV or applying for deferred prosecution make the reasons of seeking treatment different. Although our treatment modality only provided methadone maintenance, we hypothesized that self-paid heroin users choose to attend MMTP mainly for their physical discomfort suffering, especially the heroin withdrawal syndrome. This hypothesis may correspond with one study in China that reported that MMTP-related misconception (e.g., one could be completely detoxified and quit methadone treatment for the coming months) are very common among newly admitted first-time participants and misconception found at admission of MMTP predicted subsequent dropout during the treatment period [30]. That is, the heroin users dropped out the MMTP when they released their physical pain. In addition, a much higher retention rate of our publicly funded group than that of the self-paid group (18 months retention rate was 31.0 % for publicly funded and 11.0 % for the self-paid group) may also indirectly support our hypothesis. Further investigation regarding the perception and opinion toward MMTP among heroin users in Taiwan are also needed.

We found no demographic differences between heroin users under coverage of publicly funded and those of self-paid MMTP. However, QoL among publicly funded methadone patients was even worse. Although there was no demographic disparity between the publicly funded and self-paid MMTP, the publicly funded group had longer heroin abusing length and more HIV-seropositive patients than the self-paid group. This may explain the lower QoL (including physical, psychological, social, and environment QoL) of the publicly funded group than that of the self-paid group. Previous studies indicate that people who have longer heroin use or who are HIV seropositive are more likely to have worse health status and QoL than their counterparts with shorter heroin use or HIV seronegative [14, 17].

The most interesting finding is that predictors for MMTP attendance rate were different between publicly funded and self-paid groups. Our results of educational years and employment status as non-significant predictors are comparable to previous studies [31–34]. However, our results of gender as a significant predictor for publicly funded group contradict other research’s findings [31–34]. The most possible reason is the difference between publicly funded and self-paid groups. We pooled all the participants and redid a GEE analysis, and our results showed that gender cannot significantly predict attendance rate in the pooled sample (*β* = −0.054, 95 % CI = −0.124 to 0.016, *P* = 0.133). Gender difference of attendance pattern in our publicly funded group echo another recent finding [35] that persistent drug-related stigmatization paired with HIV-related discrimination among male heroin users hindered their employment and exacerbated their struggles with addiction. Although our publicly funded male participants remained on treatment, their low socio-economic status often forced them to struggle between job and regular attendance.

In addition to the demographic factors mentioned above, good social QoL and HCV seropositive were good predictors for worse and better methadone attendance, respectively, in our self-paid group. The negative influence of social QoL on the attendance rate could be explained by the misconception toward MMTP among their peer group, though we did not have solid evidences to justify this explanation, and future studies are warranted. Another explanation for the negative influence could be the good social interaction in the self-paid group. With good social interaction, the self-paid group tended to receive heroin from their friends and became less motivated to take methadone. On the other hand, the positive impact of HCV seropositive may be due to the prevalence and awareness of the self-paid patients. Methadone patients are found to have high prevalence but low awareness of HCV infection while attending MMTP [36], and their motivation to regular attendance would be enhanced through psycho-education and counseling. In contrast to the self-paid group, however, HCV seropositive had a negative influence on attendance rate of the publicly funded participants. However, because HIV infection was a covariate in the GEE model for the publicly funded group, a possibility was that the effect of HCV infection influenced by that of HIV infection. In order to clarify the role of HCV infection in the publicly funded group, we additionally did a GEE model without the covariate of HIV for the publicly funded group, and a similar result was found (*β* = −0.068, *P* = 0.012). Therefore, we tentatively concluded that attendance pattern could be different between methadone patients contracted HCV and co-morbid HIV-HCV infection. However, certain baseline variables found to have an effect on the self-paid group used to predict attendance rate may have little effect on the publicly funded MMTP group. Further studies to examine the disparity between heroin users attending publicly funded and self-paid MMTP are urgently needed for policy makers.

Although our results shed some light on the issues of QoL and treatment adherence for heroin users engaging in a MMTP, clinicians should interpret our results, including those in Tables 1, 2, and 3, in cautions because the two MMTP groups consisted of different baselines. In addition, clinicians should also understand that the publicly funded MMTP patients in Taiwan were quite different from those in the USA: the publicly funded MMTP in Taiwan currently is a kind of (partly) compulsory legal act and is not comparable with that of the Medicaid eligibility in the USA. Therefore, all publicly funded MMTP patients in this study had the situations of either with a legal issue or with HIV infection. In contrast, the self-paid MMTP patients did not have the problems that the publicly funded MMTP patients confronted. Thus, the comparisons between the two groups may be inappropriate, and our results are highly likely to be biased.

The strength of this study was that newly admitted MMTP participants with better retention were our participants, while regular attendance could indicate better treatment outcome only under the circumstance of long enough treatment retention (e.g., 1-year retention). Another advantage is that our study collected two kinds of important determinants (WHOQOL-BREF for measuring generic QoL and blood sample for confirming the chronic infectious disease) at baseline to predict attendance rate up to 18 months.

However, this study has some limitations. First, our study involved only one site, limiting the generalization of its findings. Based on this limitation, readers need to know that our comparison results between MMTP patients and Taiwan general population were biased. Second, the study employed a cross-sectional design at the intake interview and the attendance rate may be affected by some potential important ongoing or time-dependent factors. Although previous Taiwan study reported no significant difference between HIV-positive and HIV-negative methadone patients [37], it should still be interpreted with caution because our publicly funded eligibility covered all heroin users with HIV seropositive. In addition, our predictors of QoL were time-dependent factors as well. Therefore, the predictive ability of our proposed QoL factors is very likely to be changed overtime, and the predicting effect of QoL on attendance rates should be used in cautions. Third, self-stigma [38, 39], another important factor that could impact patients’ volition to attend MMT, was not measured in this study. Future studies may want to use validated questionnaire, e.g., Internalized Stigma of Mental Illness Scale [40] and Self-Stigma Scale-Short [41] to understand the impact of self-stigma on attendance rate. Fourth, some co-payment MMTPs which have had executed after the recruited period might enhance the methadone adherence among our self-paid group. Lastly, self-reported data were used. Despite we guaranteed the privacy, reporting bias may still exist.

## Conclusions

To the best of our knowledge, this is the first study comparing the QoL between publicly funded and self-paid heroin users and identifying their association of methadone attendance rate during 18 months follow-up. MMT is undoubtedly the key public health measures to control the HIV and the drug use problems. For treatment to be effective, regular attendance is necessary for reducing social costs in terms of drug-related legal and medical expenses. Previous study [42] has reported that drug users do not primarily associate QoL with health, but rather with social inclusion and self-determination. Under the circumstance of the impact of methadone maintenance was very similar in these MMTPs, if participants attend regularly. Measures to further explore what could potentially impede methadone attendance are needed. However, cautions are needed when clinicians and/or researchers interpret our results due to the different natures of our MMTP patients.

## Abbreviations

CDC: Center for Disease Control
DSM-IV: *Diagnostic and statistical manual of mental disorders, fourth edition*
GEE: generalized estimating equation
HCV: hepatitis C virus
HIV: human immunodeficiency virus
MMT: methadone maintenance treatment
MMTP: methadone maintenance treatment program
NHI: National Health Insurance
QoL: quality of life
WHOQOL-BREF: World Health Organization Quality of Life Assessment, brief version
